# Gender-Specific Associations between Perceived Neighbourhood Walkability and Meeting Walking Recommendations When Walking for Transport and Recreation for Czech Inhabitants over 50 Years of Age

**DOI:** 10.3390/ijerph110100527

**Published:** 2013-12-30

**Authors:** Jana Pelclová, Karel Frömel, Roman Cuberek

**Affiliations:** Center for Kinanthropology Research, Institute of Active Lifestyle, Faculty of Physical Culture, Palacky University in Olomouc, Tr. Miru 115, Olomouc 77111, Czech Republic; E-Mails: karel.fromel@upol.cz (K.F.); roman.cuberek@upol.cz (R.C.)

**Keywords:** ANEWS, IPAQ, walking recommendations, neighbourhood environment

## Abstract

Few studies have investigated the different effects that the built environment may have on the physical activity behaviours of men and women. Therefore, the aim of this study was to estimate the gender differences in meeting walking recommendations in relation to perceived neighbourhood walkability attributes within the active transportation and leisure-time domains for Czech inhabitants over 50 years of age. The sample included 1,417 men and 1,422 women who were randomly selected. The Abbreviated Neighbourhood Environment Walkability Scale (ANEWS) was used to obtain information about the perceived environment. The self-administered long version of the IPAQ was used to assess physical activity levels. When walking for transport, men living in neighbourhoods with high street connectivity (OR = 1.47, CI = 1.04–2.9) and higher traffic and crime safety (OR = 1.28, CI = 1.02–1.6) and women living in neighbourhoods with high proximity (OR = 1.36, CI = 1.04–1.77) and high neighbourhood aesthetics (OR = 1.36, CI = 1.04–1.76) were more likely to meet recommended levels of walking. No environmental attributes were found to significantly influence the accomplishment of walking recommendations by men or women when walking for leisure. The study results indicate the gender-specific associations between transportation-related walking and the environment factors. The consideration of those factors in the design of gender-specific walking interventions for Czech inhabitants may help the interventions to be more effective in promotion of physical activity.

## 1. Introduction

Based on ecological models of health [1], interventions on multiple levels, including environmental and policy levels, are needed to promote sufficient physical activity for health benefits. Currently, research is focused on investigating the influence that the environment might have on physical activity. Such evidence is needed to guide the development of environmental and policy initiatives that might lead to the development of physical activity-friendly neighbourhoods.

Few studies have been published regarding the different effects that the built environment may have on the physical activity behaviour of men and women [2,3,4,5]. The findings of these studies suggest that the activity-friendliness of an environment might have stronger effects on walking under certain environmental conditions and for women [5], and provide rationale for the use of models in which sex is treated as a potential moderator of the link between the perceived environment and physical activity [3]. For example Spence *et al*. [3] found out in Canadian adults, that for women, interesting scenery and the presence of many places to go within easy walking distance were significantly associated with walking at a sufficient level. However, for men, no significant associations were found between the perceived environment measures and walking. Similarly in adults from the USA, Australia and Belgium [5], the association between the walkability index and transport-related walking were stronger for women compared to men suggesting that perhaps higher levels of environmental support are needed to “encourage” women to walk for transport. Different gender patterns of environmental correlates were observed also in Japan [2]. In women, enjoyable scenery and living in urban areas were positively associated with attaining the recommended level of physical activity. Conversely to above-mentioned studies, Humpel *et al.* [6] reported neighbourhood aesthetics significantly related to neighbourhood walking in men, but not in women. Gender-specific relationship was observed between built-environment attributes of neighbourhood and sedentary behaviour as well. In Australian study [4], neighbourhood walkability was negatively associated with TV viewing time in women, but not in men. Moreover, women living in medium- and high-walkable neighbourhoods reported significantly less TV viewing time per day compared to those living in low-walkable neighbourhoods. In contrast to above-mentioned studies, a review of studies of adults [7] denoted no evidence for differences between men and women regarding the environmental determinants of physical activity and suggested further better quality explorations.

The findings of previous studies [8,9] indicate the importance of focusing on specific behaviours because the attributes of the built environment are associated with specific PA outcomes. 

Therefore, the aim of this study was to estimate the gender differences in meeting the recommended levels of walking in relation to perceived neighbourhood walkability attributes within active transportation and leisure-time domains for Czech inhabitants over 50 years of age.

## 2. Experimental Section

### 2.1. Participants and Settings

The study was carried out according to the design and methods approved by the Faculty of Physical Culture Ethics Committee at the Palacký University in Olomouc (No. 37/2012). The sample included adults and older adults from all 14 regions of the Czech Republic. A systematic random sampling from the Czech national geocoded address point database was used to identify the participants. This type of sample selection within the Czech Republic has been published previously [10]. Four thousand seven hundred and eighty-four inhabitants over 50 years of age completed the questionnaire. However, data from 1,945 respondents had to be excluded from final analysis due to incomplete data records and within-data processing according to questionnaire manuals (see below). Hence, data from 1,417 men (mean age 55.9 ± 6.7 years; mean BMI 26.6 ± 4.0 kg/m^2^) and 1,422 women (mean age 56.4 ± 6.9 years; mean BMI 25.6 ± 4.0 kg/m^2^) were included in the final analysis. All inhabitants who agreed to participate provided verbal and written consent. This study was carried out from 2006 to 2011. The questionnaire data investigated physical activity during the previous seven days. The weather might influence physical activity in adults and older adults [11]; thus, the data were intentionally collected during moderate spring and autumn seasons having mean temperatures of ≈10 °C (according to measurements taken four times within 24-hour periods), avoiding warm summers and cold winters.

### 2.2. Measurements

#### 2.2.1. Neighbourhood Environment

Modified and culturally adapted abbreviated versions of the questionnaire entitled “Neighbourhood Environment Walkability Scale” (ANEWS) [12] were used to obtain information about the perceived environment. ANEWS is an instrument that assesses the perception of neighbourhood design features related to physical activity, including residential density, land use mix (including both indices of proximity and accessibility), street connectivity, infrastructure for walking/cycling, neighbourhood aesthetics, and traffic and crime safety. 

#### 2.2.2. Physical Activity

The self-administrative long version of the “International Physical Activity Questionnaire” (IPAQ) was used to assess physical activity levels. The reliability and validity of the instrument has been tested across 12 countries [13]. The Czech-translated version complied with standardised translating guidelines including back-translation into English [14] and has been used in previous Czech research [10,15]. The IPAQ long version investigated walking, moderate PA and vigorous PA in four life domains: work-related (paid jobs, farming, and voluntary jobs), house and gardening work (outside and inside the home), leisure time (recreational and sport activities) and active transportation. In this study with regard to populations over 50 years of age, only walking with a cut-off time of 30 min five times a week [16] within the active transportation and leisure time domains was assessed.

### 2.3. Data Analysis

The results of physical activity were processed according to the guidelines of the “IPAQ Research Committee” [14]. Data assessments of the perceived neighbourhood environment were processed according to the scoring for Neighborhood Environment Walkability Scale—Abbreviated [17]. The items in the factors were summed to provide a total score for these environmental categories: land use mix—proximity, land use mix—accessibility, street connectivity, infrastructure for walking/cycling, neighbourhood aesthetics, and traffic and crime safety. These summed scores were then divided by the number of items in each category. In case of residential density, the items were multiplied by coefficients and summed. The final scores of all categories were transformed into categorical variables with two levels: low and high perception of the environment. The cut-off points for these levels were set according to median of the sample (land use mix—proximity: low <3, high ≥3; land use mix accessibility: low ≤3, high >3; street connectivity: low ≤3, high >3; infrastructure for walking/cycling: low ≤2.5, high >2.5; neighbourhood aesthetics: low ≤2.5, high >2.5; traffic and crime safety: low ≥2, high <2; residential density: low <146, high ≥146.

The statistical package SPSS 18 (SPSS, Inc., Chicago, IL, USA) was used to statistically process the data. The significance level was set at *p* < 0.05. Binary logistic regression (enter method) was used separately for gender (men and women) for dichotomous outcomes: reaching the recommended levels of walking for transportation and walking for leisure as dependent variables. The independent variables entered into the binary logistic regression were: residential density, land use mix (including both indices of proximity and accessibility), street connectivity, infrastructure for walking/cycling, neighbourhood aesthetics, traffic and crime safety. The models have been controlled for age, education and BMI. The first group in each category was the referent group in each binary logistic regression analysis.

## 3. Results

In this study, significantly more women (67.3%) than men (60.7%) accomplished the recommendation of 30 min of walking five times a week (χ^2^ = 13.45, *p* < 0.001). Similarly when walking for transportation, significantly higher proportion of women (51.8%) than men (40%) met the recommendation (χ^2^ = 37.53, *p* < 0.001). The differences between men (21.2%) and women (22.3%) were not significant, when walking for recreation (χ^2^ = 0.52, *p* = 0.47). 

Table 1 presents the association between perceived neighbourhood walkability attributes and meeting the recommendations of walking for transportation separately for men and women. In men, two environmental attributes were found to be significantly related to meeting walking recommendations. Men living in neighbourhoods with high street connectivity and with higher traffic and crime safety were more likely to meet the recommended levels when walking for transport. In women, also two significant environmental attributes were found. Women living in neighbourhoods with high proximity and women living in areas with high neighbourhood aesthetics were more likely to meet walking recommendations when walking for transport.

**Table 1:** Gender-specific associations between perceived neighborhood walkability and meeting the recommendation of walking for transportation.

Neighbourhood Walkability Attributes	Meeting Recommendations When Walking for Transportation			
	Men		Women	
	n (%)	OR (CI)	n (%)	OR (CI)
**Residential density**				
low	221		280	
	(37.4)		(48.1)	
high	350	1.25	456	1.17
	(42.4)	(0.98–1.61)	(54.3)	(0.93–1.48)
**Land use-mix-proximity**				
low	144		173	
	(37.1)		(44.7)	
high	427	111	563	1.36 *
	(41.5)	(0.84–1.47)	(54.4)	(1.04–1.77)
**Land use-mix-accessibility**				
low	3		4	
	(33.3)		(54.1)	
high	568	1.09	732	0.8
	(40.3)	(0.27–4.45)	(51.7)	(0.17–3.72)
**Street connectivity**				
low	60		81	
	(32.1)		(32.1)	
high	511	1.47 *	511	0.85
	(41.5)	(1.04–2.9)	(41.5)	(0.59–1.21)
**Infrastructure for walking/cycling**				
low	65		65	
	(42.2)		(51.3)	
high	506	0.7	655	1.19
	(40.1)	(0.48–1.02)	(51.8)	(0.81–1.76)
**Neighbourhood aesthetics**				
low	91		59	
	(37.9)		(43.7)	
high	480	1.23	677	1.36 *
	(40.8)	(0.92–1.66)	(52.6)	(1.04–1.79)
**Traffic and crime safety**				
low	261		345	
	(37.5)		(49.7)	
high	310	1.28 *	391	1.01
	(43.0)	(1.02–1.60)	(53.7)	(0.88–1.37)

Notes: OR—odds ratios; CI—confidence interval; * Statistical significance (*p* < 0.05); The model has been controlled for age, BMI and education.

Table 2 shows the gender associations between perceived neighbourhood walkability attributes and meeting recommended levels of walking for leisure. No environmental attributes were found to be significantly associated with the accomplishment of walking recommendations for men or women.

**Table 2:** Gender-specific associations between perceived neighbourhood walkability and meeting the recommendation of walking for leisure.

Neighbourhood Walkability Attributes	Meeting Recommendations When Walking for Leisure			
	Men		Women	
	n (%)	OR (CI)	n (%)	OR (CI)
**Residential density**				
low	117		129	
	(19.8)		(22.2)	
high	183	1.25	188	1.04
	(22.2)	(0.92–1.68)	(22.4)	(0.76–1.33)
**Land use-mix-proximity**				
low	83		90	
	(21.4)		(23.3)	
high	217	0.96	227	0.91
	(21.1)	(0.69–1.35)	(21.9)	(0.66–1.25)
**Land use-mix-accessibility**				
low	3		1	
	(33.3)		(14.3)	
high	279	0.49	316	1.88(0.22–16.03)
	(21.1)	(0.12–1.99)	(22.3)	
**Street connectivity**				
low	39		33	
	(20.9)		(20.9)	
high	261	1.1	284	1.08
	(21.2)	(0.73–1.66)	(22.5)	(0.70–1.66)
**Infrastructure for walking/cycling**				
low	35		25	
	(22.7)		(18.5)	
high	265	0.82	292	1.23
	(21.0)	(0.53–1.28)	(22.7)	(0.76–1.99)
**Neighbourhood aesthetics**				
low	46		54	
	(19.2)		(18.9)	
high	254	1.27	263	1.31
	(21.6)	(0.88–1.84)	(23.2)	(0.93–1.84)
**Traffic and crime safety**				
low	143		151	
	(20.6)		(21.8)	
high	157	1.12	166	1.07
	(21.8)	(0.85–1.46)	(22.8)	(0.82–1.39)

Notes: OR—odds ratios; CI—confidence interval; * Statistical significance (*p* < 0.05); The model has been controlled for age, BMI and education.

## 4. Discussion

In this cross-sectional study, the gender differences in meeting walking recommendations in relation to perceived neighbourhood walkability attributes were investigated. More women than men accomplished the recommended levels of walking. This finding supports the previous study of Czech inhabitants [10] in which men were shown to be more likely than women to meet the guidelines for moderate and vigorous physical activity, but were less likely to walk for 30 min five times per week. Gender differences in the accomplishment of walking recommendations were found between the active transportation and leisure time domains. Whereas within active transportation, women were more likely than men to meet the recommendation (40% *vs.* 51.8%), when walking for recreation, no differences were found (21.2% *vs.* 22.3%). 

Gender differences were found in terms of the perceived neighbourhood walkability attributes related to meeting walking recommendations when walking for transport. For men, living in neighbourhoods with high street connectivity and with higher traffic and crime safety was positively associated with meeting the recommendations. However, for women, the likelihood of meeting walking recommendations was related to living in neighbourhoods with high proximity to different destinations and high neighbourhood aesthetics. Similarly, results from previous studies investigating the association between perceived neighbourhood environmental attributes and transport-related walking in the USA, Australia and Belgium [5] indicated gender differences in which a stronger association was found for women. However, this association was not identified across all three countries. Similarly, Japanese men and women were found to have different patterns of social, psychological and environmental correlations [2]. Among environmental correlations, enjoyable scenery was positively associated and living in rural areas was negatively associated with meeting the Japanese physical activity level recommendations in women. On the other hand, a review of studies of the environmental determinants of physical activity in adults [7] suggested that possible associations with the availability of sidewalks and environmental aesthetics may be potential determinants of walking behaviour among men, but not among women. Similarly in Australian study [6], neighbourhood aesthetics was significantly related to neighbourhood walking in men. The results of these studies confirm the gender-specific association between the environment and transport-related walking. However, inconsistent results from different studies suggest that gender differences may vary across countries and justify additional research. 

Gender differences were not found while investigating the relationship between meeting walking recommendations and the perceived walkability attributes when walking for recreation. Regardless of gender, no environmental attributes were found to be significantly associated with the accomplishment of walking recommendations. These findings support the review of studies of the relationship between the built environment and physical activity among adults [18]. In this study, the built environment was more likely to be associated with transportation-related walking compared to other types of physical activity including recreational walking. Moreover, another review of studies of child, adult and older adult populations [19] summarising the correlation between the built environment and walking noted that there was a positive relationship between some environmental attributes and walking for transportation; however, results regarding recreational walking were less clear. 

The findings of this study fill the research gap in exploring the gender-specific association between the environment and physical activity [5,7]. However, this study has some limitations. First, the study was cross-sectional in design, and did not allow for the identification of any causal relationships. Second, the neighbourhood environment was assessed subjectively. Several previous studies [20,21,22] showed poor agreements between objective and perceived measures of the built environment. Thus, objective measures of the built environment may have their own distinctive effects on people’s health and well-being, and they are worthwhile to be investigated.

## 5. Conclusions

In summary, the findings of this study contribute to the research evidence of PA and environmental association due to specific Czech environmental, cultural and historical conditions. The study results indicate the gender-specific associations particularly between transportation-related walking and environmental factors. The findings of this study suggest, that the design of walking interventions programmes for Czech inhabitants 50 years of age and older that are gender-specific and account for those environment factor may be more effective in promotion of physical activity. For potential urban planning and public health initiatives, neighbourhood aesthetics, land use mix-proximity, street connectivity and neighbourhood safety might be taken into consideration.

## References

[1] Sallis J.F., Owen N., Fisher E.B., Glanz K., Rimer B.K., Viswanth K. (2008). Ecological models of health behavior. Health Behavior and Health Education: Theory, Research, and Practice.

[2] Shibata A., Oka K., Harada K., Nakamura Y., Muraoka I. (2009). Psychological, social, and environmental factors to meeting physical activity recommendations among Japanese adults. Int. J. Behav. Nutr. Phys. Act..

[3] Spence J.C., Plotnikoff R.C., Rovniak L.S., Ginis K.M., Rodgers W., Lear S.A. (2006). Perceived neighbourhood correlates of walking among participants visiting the Canada on the Move website. Can. J. Public Health.

[4] Sugiyama T., Salmon J., Dunstan D.W., Bauman A.E., Owen N. (2007). Neighborhood walkability and TV viewing time among Australian adults. Amer. J. Prev. Med..

[5] Van Dyck D., Cerin E., Conway T.L., de Bourdeaudhuij I., Owen N., Kerr J., Cardon G., Frank L.D., Saelens B.E., Sallis J.F. (2012). Perceived neighborhood environmental attributes associated with adults transport-related walking and cycling: Findings from the USA, Australia, and Belgium. Int. J. Behav. Nutr. Phys. Act..

[6] Humpel N., Owen N., Iverson D., Leslie E., Bauman A. (2004). Perceived environment attributes, residential location, and walking for particular purposes. Amer. J. Prev. Med..

[7] Wendel-Vos W., Droomers M., Kremers S., Brug J., van Lenthe F. (2007). Potential environmental determinants of physical activity in adults: A systematic review. Obes. Rev..

[8] Giles-Corti B., Timperio A., Bull F., Pikora T. (2005). Understanding physical activity environmental correlates: Increased specificity for ecological models. Exerc. Sport Sci. Rev..

[9] Owen N., Humpel N., Leslie E., Bauman A., Sallis J.F. (2004). Understanding environmental influences on walking: Review and research agenda. Amer. J. Prev. Med..

[10] Frömel K., Mitáš J., Kerr J. (2009). The associations between active lifestyle, the size of a community and SES of the adult population in the Czech Republic. Health Place.

[11] Belza B., Walwick J., Schwartz S., LoGerfo J., Shiu-Thornton S., Taylor M. Older Adult Perspectives on Physical Activity and Exercise: Voices from Multiple Cultures. http://www.ncbi.nlm.nih.gov/pmc/articles/PMC1277949/#__ffn_sectitle.

[12] Cerin E., Saelens B.E., Sallis J.F., Frank L.D. (2006). Neighborhood environment walkability scale: Validity and development of a short form. Med. Sci. Sport. Exercise.

[13] Craig C.L., Marshall A.L., Sjostrom M., Bauman A.E., Booth M.L., Ainsworth B.E., Pratt M., Ekelund U., Yngve A., Sallis J.F., Oja P. (2003). International physical activity questionnaire: 12-country reliability and validity. Med. Sci. Sport. Exercise.

[14] International Physical Activity Questionnaire Cultural Adaptation. http://www.ipaq.ki.se/cultural.htm.

[15] Pelclová J., Vašíčková J., Frömel K., Djordjević I. (2009). Leisure time, occupational, domestic, and commuting physical activity of inhabitants of the Czech Republic aged 55–69: Influence of socio-demographic and environmental factors. Acta Univ. Palackianae Olomucensis. Gymnica.

[16] Haskell W.L., Lee I.M., Pate R.R., Powell K.E., Blair S.N., Franklin B.A., Macera C.A., Heath G.W., Thompson P.D., Bauman A. (2007). Physical activity and public health: Updated recommendation for adults from the American college of sports medicine and the American heart association. Circulation.

[17] Sallis J.F. Scoring for the Neighborhood Environment Walkability Scale—Abbreviated (NEWS-A). http://sallis.ucsd.edu/Documents/Measures_documents/NEWS_A_scoring.pdf.

[18] McCormack G.R., Shiell A. (2011). In search of causality: A systematic review of the relationship between the built environment and physical activity among adults. Int. J. Behav. Nutr. Phys. Act..

[19] Saelens B.E., Handy S.L. (2008). Built environment correlates of walking: A review. Med. Sci. Sport. Exercise.

[20] Ball K., Jeffery R.W., Crawford D.A., Roberts R.J., Salmon J., Timperio A.F. (2008). Mismatch between perceived and objective measures of physical activity environments. Prev. Med..

[21] Brownson R.C., Hoehner C.M., Day K., Forsyth A., Sallis J.F. (2009). Measuring the built environment for physical activity: State of the science. Amer. J. Prev. Med..

[22] Hoehner C.M., Ramirez L.K.B., Elliott M.B., Handy S.L., Brownson R.C. (2005). Perceived and objective environmental measures and physical activity among urban adults. Amer. J. Prev. Med..

